# Metabolic profiling of adolescent non-alcoholic fatty liver disease

**DOI:** 10.12688/wellcomeopenres.14974.2

**Published:** 2019-09-19

**Authors:** April Hartley, Diana L. Santos Ferreira, Emma L. Anderson, Debbie A. Lawlor

**Affiliations:** 1 Musculoskeletal Research Unit, Translational Health Sciences, University of Bristol, Bristol, BS10 5NB, UK; 2 MRC Integrative Epidemiology Unit, University of Bristol, Bristol, BS8 2BN, UK; 3 Population Health Sciences, Bristol Medical School, University of Bristol, Bristol, BS8 2BN, UK

**Keywords:** Metabolomics, ALSPAC, Steatosis, NAFLD, Lipoproteins

## Abstract

**Background: **Adolescent non-alcoholic fatty liver disease (NAFLD) is associated with cardiometabolic risk factors. The association between adolescent NAFLD and a wide range of metabolic biomarkers is unclear. We have attempted to determine the differences in metabolic profile of adolescents with and without markers of NAFLD.

**Methods: **We performed cross-sectional analyses in a sample of 3,048 participants from the Avon Longitudinal Study of Parents and Children at age 17. We used three indicators of NAFLD: ALT >40 U/l; AST >40 U/l and ultrasound scan-assessed steatosis. Associations between each measure of NAFLD and 154 metabolic traits, assessed by Nuclear Magnetic Resonance, were analyzed by multivariable linear regression, adjusting for age, sex and BMI.

**Results: **All three indicators of NAFLD were associated with ~0.5 standard deviation (SD) greater concentrations of all extremely large to small very low-density lipoproteins (VLDL) measures. ALT >40U/l was associated with ~0.5SD greater concentrations of very small VLDLs, intermediate-density lipoproteins and low-density lipoproteins. Concentrations of most cholesterols, including remnant cholesterol, all triglycerides and monounsaturated fatty acids, in addition to glycoprotein acetyls (inflammatory marker), were also higher in participants with NAFLD.

**Conclusions: **We have identified differing metabolic profiles between adolescents with and without indicators of NAFLD. These results provide the foundations for future research to determine whether these differences persist and result in adverse future cardiometabolic health.

## Introduction

Non-alcoholic fatty liver disease (NAFLD) is the most common chronic liver disease in adolescents ^1^, with an estimated prevalence of 7.6% in the general population, increasing to 34.2% in obese populations ^2^. NAFLD occurs when at least 5% of hepatocytes are infiltrated by fat, in those without significant alcohol consumption (<20g/day alcohol for women and <30g/day for men) ^3^. NAFLD encompasses both steatosis (accumulation of fat in the liver), without evidence of hepatocyte injury, and steatohepatitis (NASH), with hepatocyte injury and inflammation, which can progress to cirrhosis and hepatocellular carcinoma (HCC) ^4, 5^.

The relationship of multiple metabolic pathways, including those related to lipids, fatty acids, glycolysis and insulin resistance, with NAFLD is complex, as these may be part of a ‘multiple-hit’ process that results in hepatic steatosis ^6^, or a consequence of NAFLD, and mediators of the effect of NAFLD on subsequent adverse cardiometabolic outcomes ^7^. In relation to the latter, in adults NAFLD is associated with adverse cardiometabolic risk factors, including insulin resistance, diabetes, and dyslipidemia ^3, 8– 10^ and is therefore described as the hepatic component of the metabolic syndrome ^1, 11^. Using a population of adolescents from the Avon Longitudinal Study of Parents and Children (ALSPAC), we recently showed that, even in unselected (and otherwise healthy) adolescents, ultrasound scan-determined hepatic steatosis is associated with higher levels of fasting triglycerides, glucose and insulin, and reduced levels of HDL cholesterol (HDLc), independent of body mass index (BMI) or total body fat mass (TBFM) ^12^.

Given the role of the liver in multiple metabolic pathways, it is plausible that adolescent NAFLD is associated with a much wider range of circulating metabolomic biomarkers than standard laboratory lipid (triglycerides and HDL/LDL cholesterol) and glucose measurements. Identifying whether this is the case could provide valuable measures of disease progression, in particular identifying potential mediators of a possible effect of NAFLD in early life (adolescence) on subsequent adverse cardiometabolic outcomes. Specifically, if we find widespread metabolic disruption in adolescents with NAFLD, this could indicate those at increased risk of future adverse cardiometabolic outcomes such as type 2 diabetes or cardiovascular disease. Previous metabolomics studies of NAFLD have largely had small sample sizes (N=30 to N=467) ^13– 19^, and have been undertaken in select populations, such as obese individuals (the greatest risk factor for NAFLD) ^15, 17– 19^. One exception is a recent study by Kaikkonen *et al.* of unselected (healthy) European adults (mean age 42 years) from the Young Finns cohort (N=1,575), which examined the association of a range of metabolic biomarkers (using the same Nuclear Magnetic Resonance (NMR) platform that we have used in this study) with subsequent occurrence of NAFLD. They found that increased concentrations of all very low-density lipoprotein (VLDL) particle concentrations, except very small VLDL particles, and triglycerides in VLDLs, as well as several other metabolic traits, were associated with subsequent development of fatty liver (odds ratio per 1SD increase in log transformed metabolite ranged from 1.38 to 1.51 for the VLDL particle concentrations) ^20^.

To the best of our knowledge, no study has yet examined the differences in metabolic profile (the collection of lipids and low molecular weight metabolites) of adolescents with NAFLD, in whom the prevalence of, and years of exposure to risk factors, including alcohol, for NAFLD is lower than in adults. We therefore aimed to determine whether there was a difference in metabolic profile between adolescents with and without NAFLD, using three indicators of NAFLD. Although our study is cross-sectional, and therefore cannot determine the direction of associations, we were interested in the potential that differences in these profiles in adolescents could influence future progression and subsequent adverse cardiometabolic outcomes. Therefore, we hypothesized that NAFLD would precede metabolic disruption and treated indicators of NAFLD as the exposures in our analyses.

## Methods

### Study population

We performed cross-sectional analysis in a population of adolescents from ALSPAC. ALSPAC is a prospective cohort of 14,541 pregnancies with an expected delivery date between 01/04/1991 and 31/12/1992, recruited from the former Avon area, Southwest England ^21, 22^. Of these 14,541 pregnancies, there were 14,062 live births ^21^. The cohort has been followed up repeatedly since birth, including questionnaires and clinical assessment from age 7 years ^19^. When the original children were 7 years old, an attempt was made to increase the sample size, resulting in the addition of 706 pregnancies and 713 children who were alive at one year ^22^. The study website contains details of all the data that is available through a fully searchable data dictionary: http://www.bristol.ac.uk/alspac/researchers/data-access/data-dictionary/.

Ethical approval for the study was obtained from the ALSPAC Ethics and Law committee and the local Research Ethics Committees. All data was collected and managed following approved protocols. The main caregiver initially provided consent for child participation and from age 16 years the offspring themselves have provided written informed consent. 10,101 (66%) adolescents were still eligible for invite to the research clinic at age 17.

### Assessment of NAFLD

***Ultrasound (USS) assessment of steatosis.*** Details of the USS assessment in ALSPAC have been published previously ^12^. Briefly, at the 17- to 18-year follow-up clinic, a pseudo-random sample (selected based on attendance of clinic between 2008 and 2011- the period when we had access to staff and resources for this substudy) of 1,887 individuals underwent USS of the upper abdomen by one of four trained sonographers using a Siemens Acuson S2000 USS system. Liver fat was assessed by echogenicity during deep inspiration and recorded as present, absent or uncertain following an established protocol ^23, 24^. There was a high level of agreement between sonographers, assessed immediately after training and at six-month intervals (absolute agreement >98%). There was no evidence of these participants ever experiencing jaundice, hepatitis or any other liver disease. None of the participants were taking medications suggestive of liver disease or known to influence liver function.

***Blood-based biomarkers.*** Fasting (overnight or minimum of 6-hours) blood samples were immediately spun and frozen at -80°C. Measurements were assayed between three and nine months after samples were taken, with no previous freeze-thaw cycles. All assays were completed in the same laboratory at the University of Glasgow. Alanine aminotransferase (ALT) and aspartate aminotransferase (AST) were measured by automated analyzer with enzymatic methods. We used a threshold of >40U/l of ALT/AST as an indicator of NAFLD, as this was the most commonly used threshold in adolescent studies based on a recent systematic review ^2^.

### NMR metabolic profiling

Metabolic profiling was performed using a high-throughput proton NMR platform on fasting plasma samples collected at the clinic. The 154 metabolic traits included in this analysis represent a broad molecular signature of systemic metabolism and includes absolute concentrations of 14 lipoprotein subclasses (particle concentration, lipid concentrations and composition), fatty acids and fatty acid compositions, amino acids, ketone bodies, glycolysis and gluconeogenesis-related metabolic traits ^25– 27^. The method is based on three molecular windows: LIPO identifies lipoprotein subclasses and apolipoproteins, low molecular weight molecule (LMWM) identifies amino acids, glucose, glycolysis-related metabolites, glycoproteins and ketone bodies and LIPID identifies serum lipid constituents such as unsaturated and saturated fatty acids ^26, 27^. The protocol is published elsewhere ^25– 28^.

14 lipoprotein subclasses were identified from this platform ^26, 27^. Very low-density lipoproteins (VLDL) were subdivided into six subclasses based on particle size, from extremely large VLDL with particle diameters of above 75.0nm (with a possible contribution of chylomicrons) to very small with average particle diameters of 31.3nm. Very large, large, medium and small had average diameters of 64.0nm, 53.6nm, 44.5nm and 36.8nm, respectively. Intermediate density lipoproteins (IDLs) had a mean particle diameter of 28.6nm. The three low density lipoprotein subclasses (large, medium and small) had average diameters of 25.5nm, 23.0nm and 18.7nm, respectively. There were four classes of high density lipoproteins (HDLs) identified, including very large with an average diameter of 14.3nm, large with an average diameter of 12.2nm, medium with a diameter of 10.9nm and small with a diameter of 8.7nm. The mean sizes for VLDL, LDL and HDL particles were calculated by weighting the corresponding subclass diameters with their particle concentrations. Seven traits were determined for each lipoprotein subclass: particle concentration, total lipids, total cholesterol, phospholipids, triglycerides, free cholesterol and esterified cholesterol. Remnant cholesterol was defined as non-HDL and non-LDL cholesterol (i.e. VLDL and IDL cholesterol). Fatty acids (FAs) were measured as absolute concentrations and as percentage of total FAs. There is a high analytical consistency, in epidemiological settings, between metabolic measures quantified by the NMR metabolomics platform and the concentrations obtained from routine clinical chemistry and other analytical methods, such as gas chromatography ^29, 30^. In addition, the consistency of biomarker associations with disease incidence for metabolic traits quantified by NMR and two widely used mass spectroscopy platforms has been demonstrated ^29, 30^.

### Other outcomes

We compared associations with standard clinical chemistry assessments of lipids (total cholesterol, triglycerides, LDLc and HDLc), glucose, insulin and C-reactive protein (CRP). Plasma lipids (total cholesterol, HDLc and triglycerides) were assessed by modification of the standard Lipid Research Clinics Protocol using enzymatic reagents for lipid determination. The Friedewald equation was used to calculate LDLc (LDLc= total cholesterol – (HDLc+[0.45 × triglycerides])) ^31^. Insulin was assessed by ELISA (Mercodia) that does not cross-react with proinsulin and plasma glucose by the automated enzymatic (hexokinase) method. Plasma glucose and CRP were measured by an automated assay. All inter- and intra- CVs for these blood assays were <5%. We have previously reported associations of USS-assessed steatosis (USS steatosis) with these measurements in this cohort ^12^, but present them here in the sub-sample of participants with NMR data as a test of agreement between the two analytical approaches. It also enables us to examine associations of insulin and CRP alongside the wide range of NMR metabolic traits that are the focus of this paper.

### Covariables

After removing those with high alcohol consumption (see below), we considered sex, age and BMI to be the primary confounders in our analyses. Sex was recorded at birth and age at the clinic calculated from birth and clinic date. BMI was assessed at the clinic at age 17 as weight (kg)/height (m ^2^). Height was measured using a Harpenden stadiometer to the last complete millimetre. Weight was assessed using a Tanita Body Fat Analyser (Model TBF 401A) and was measured to the nearest 50g.

**Figure 1.**
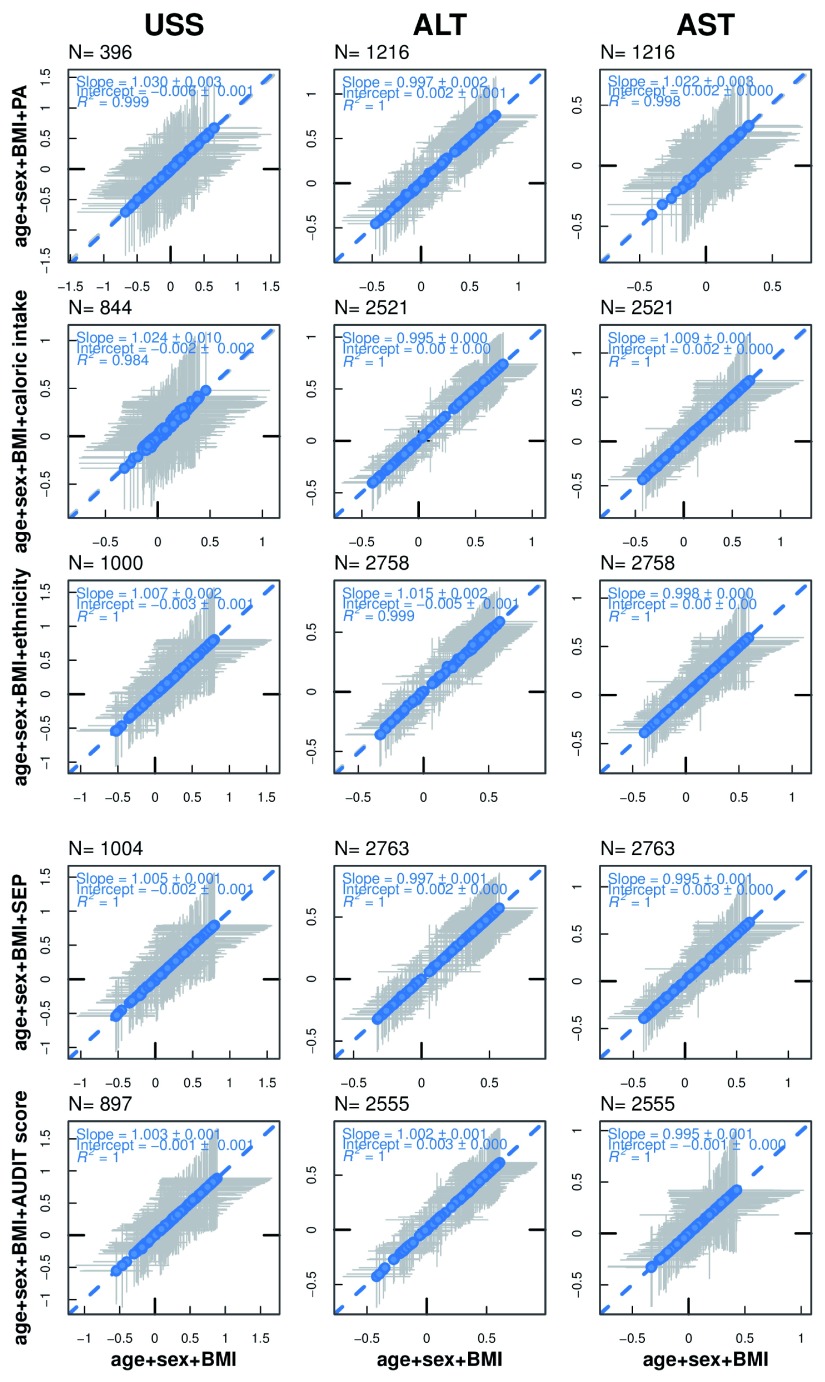
Correspondence between models adjusted for age, sex and BMI and models adjusted for age, sex, BMI and additional confounders for each indicator of NAFLD. Each point represents a metabolic trait and the position represents the difference in mean metabolic trait concentrations in standard deviation units adjusted for age, sex and BMI (x-axis) and difference in mean metabolic trait concentrations in standard deviation units adjusted for age, sex, BMI and an additional confounder (y-axis). Horizontal grey lines represent confidence intervals for x-axis associations, and vertical grey lines represent confidence intervals for y-axis associations. The blue dashed line represents the regression line between x-axis and y-axis models for each metabolic trait. R ^2^ indicates goodness-of-fit. A slope of 1 with an intercept of 0 (grey dashed line) and all dots sitting on that line (R ^2^ = 1) would indicate exact correspondence, in terms of direction and magnitude, between associations of the two models and therefore addition of the confounder to the model does not change magnitude or direction of associations. Abbreviations: AST: aspartate aminotransferase; ALT: alanine aminotransferase, USS: ultrasound scan; PA: physical activity; SEP: socioeconomic position; AUDIT: alcohol use disorders identification test score; BMI: body mass index.

Our a priori assumption was that BMI would be the main confounder of the associations of interest, but we explored the potential impact of adjusting for additional confounders (physical activity, caloric intake, ethnicity, maternal education and AUDIT score), by comparing results from linear regression analyses with adjustment for age, sex and BMI (model 2) to those with additional adjustment for each of these confounders in those with data for the specific confounder ( Figure 1).

Physical activity (PA) was assessed at average age 15 using an Actigraph PA monitor (Manufacturing Technology Incorporated, Fort Walton Beach, Florida) worn for one week. Data was determined to be valid if the monitor was worn for at least 10 hours on at least three days. Average minutes of moderate to vigorous intensity PA (MVPA) per valid day was calculated based on a value of greater than 3600 counts per minute to define MVPA ^32, 33^. The full protocol for PA assessment is described elsewhere ^34^. Caloric intake at average age 13 (kcal) was assessed by self-completed food diary (with parental help). Methodology for dietary assessment is described in detail elsewhere ^35^. Ethnicity and maternal highest educational attainment were self-reported by questionnaire during pregnancy. Socioeconomic position was determined using maternal education assessed around the time of the participant’s birth. Total body fat mass (TBFM, grams) was assessed by dual x-ray absorptiometry (DXA), using a Lunar prodigy narrow fan beam densitometer (GE Healthcare).

### Eligibility criteria

**Figure 2.**
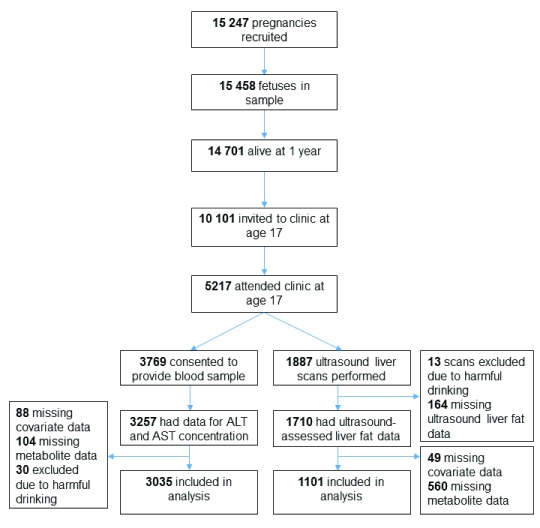
Flowchart of recruitment from original birth cohort to study populations for these analyses. Abbreviations: ALT: alanine aminotransferase; AST: aspartate aminotransferase.

Participants’ alcohol consumption was assessed at mean age 16.7 and 17.8 years using the Alcohol Use Disorders Identification Test (AUDIT) ^36^. A score of 16 or greater at both time points indicated harmful drinking. Thirteen participants were removed from the USS dataset and 30 individuals were removed from the AST/ALT dataset based on these criteria. After removal of these individuals, 3,035 participants had data on ALT and AST concentrations, metabolic traits and covariates and 1,101 had data on USS steatosis, metabolic traits and covariates ( Figure 2 shows the participant flow).

### Statistical analysis

We analyzed differences in concentrations of each metabolic trait (treated here as the outcome) between those individuals with and without NAFLD, as defined by each of the three indicators, using multivariable linear regression models, with model 1 adjusted for sex and age at assessment and model 2 additionally adjusted for BMI (modelled as a continuously measured variable) at assessment. For our main analyses we treated each of the three indicators as separate exposures such that for each one we compare differences in metabolic traits between those with the indicator and those without, even if those without one of the indicators (e.g. USS steatosis) had one of the other indicators (e.g. high ALT). As several metabolic traits displayed a skewed distribution, we performed our regression with robust standard errors (SE). We performed our analyses with the metabolites in standard deviation [SD] units and also in molar concentrations (mostly μmol/l). The former are presented in the main paper and allow us to directly compare associations across all metabolic traits as they are all in the same (SD) units. The latter are provided in extended data tables and provide outcomes for each trait in clinically meaningful units. Analyses were performed using Stata version 14.2 (Stata corp., Texas, USA) and R version 3.2.2 (R statistical computing, Vienna, Austria).

As recommended by the American Statistical Association and other bodies, we focus our interpretation of the results on the size of associations and their confidence intervals (CIs), rather than arbitrary *p*-value thresholds ^37^. However, we do indicate those with a multiple-testing corrected *p-*value so that the role of chance can be considered. To account for multiple testing and the correlation between metabolic traits, we determined our *p-*value threshold based on principal component analysis using all 154 standardized metabolic traits ^28, 38, 39^. The threshold was defined as α/ *A*, where *A* is the number of principal components (PCs) which explain at least 95% of the variance in standardized metabolic data and *α*=0.05. 16 PCs explained at least 95% of the variance, so our threshold was *p*<0.05/16= 0.003, which is equivalent to *p*<0.05 after accounting for multiple testing.

### Sensitivity and additional analyses

We repeated analyses after removing those who stated they had diabetes at the clinic from our analyses (n=10 for models including AST or ALT, n=7 for USS). The reason for this sensitivity analysis was that we thought it possible that a diagnosis of diabetes and its treatment might be a confounder of our associations, but with so few participants with this condition a conventional multivariable regression may not fully adjust for this. We also removed individuals who were at least seven SD above or below the mean value for a metabolic trait to determine the influence of potential outliers. We repeated analysis using enzyme-assessed measures of triglycerides, LDLc, HDLc and glucose to check that effect sizes agreed between the two methods of quantification (enzymatic and NMR). In addition, we adjusted for DXA determined TBFM instead of BMI. Whilst in our previous analyses of the impact of USS steatosis on conventional lipids (LDLc, HDLc and triglycerides), glucose and insulin we found no difference in the impact of adjustment for BMI or TBFM (a more direct measure than BMI of fat), we wanted to check that that was also the case here with a much greater range of metabolic outcomes.

As noted in the *Introduction*, it is possible that the metabolic traits we have assessed are altered prior to liver fat accumulation; they may be causes and/or predictors of risk. Indeed, one previous large general cohort study examined the cross-sectional association of the same NMR metabolic traits with USS steatosis but assumed that steatosis was the outcome ^17^, rather than our hypothesis that NAFLD might result in subsequent metabolic disruption. We therefore performed sensitivity analyses to explore the associations of fasting enzyme-assessed triglycerides, LDLc, HLDc and glucose assessed at age 15 with subsequent NAFLD at age 17 using logistic regression adjusted for age, sex and BMI. Fasting blood samples were collected, and BMI assessed, at age 15 using the same methodology and protocols as at age 17. The clinical laboratory assessment of lipids and glucose were also assessed using the same methods as those used at the 17 year clinic ^12^. 648 individuals with USS data and 2,034 with ALT/AST levels at age 17 had data for enzyme-assessed lipids and glucose at age 15.

We repeated our main analyses with a consistent ‘control’ group across all three indicators – i.e. for each indicator we compared those having the indicator to those who did not have any of the three indicators – in order to make sure that any differences were not biased towards the null because of some of those without a particular indicator having some hepatic steatosis as suggested by one of the other indicators. 1,022 individuals with data for ALT/AST and USS did not meet any criteria for NAFLD and formed the control group for these analyses.

At the suggestion of one of the reviewers we explored the association of a genetic variant (rs738409 C >G SNP (encoding PNPLA3 I148M)) that has been shown to be a robust predictor of CT-assessed NAFLD ^40– 42^ with our USS and ALT and AST markers of NAFLD using logistic regression. These analyses were considered exploratory due to limited power, given the SNP explains just 2.4% of the variance in CT NAFLD and we only had small sample sizes (participants with genetic and outcome data) for these analyses (N=2,242 for ALT/AST, N=794 for USS).

## Results

Descriptive characteristics of those with USS data for steatosis and those with ALT/AST data are summarized in Table 1. 44.2% of the USS population and 48.1% of the ALT/AST population were male. The mean age of individuals included in our analyses was 17.9 (SD 0.4) years for the USS population and 17.8 (SD 0.4) years at assessment for the ALT/AST population.

**Table 1.** Characteristics of the study populations.

	ALT/AST population		USS population	
	N (total)	N (%)	N (total)	N (%)
Sex (male)	3035	1461 (48.1)	1101	487 (44.2)
USS steatosis	1088	25 (2.3)	1101	25 (2.3)
Elevated ALT (>40U/l)	3035	91 (3.0)	1088	33 (3.0)
Elevated AST (>40U/l)	3035	68 (2.2)	1088	29 (2.7)
Maternal education	2763		1004	
*CSE/Vocational*		487 (17.6)		196 (19.5)
*O Level*		894 (32.4)		332 (33.1)
*A Level and above*		1382 (50.0)		476 (47.4)
Ethnicity (White British)	2758	2700 (97.9)	1000	980 (98.0)
	**N**	**Mean (SD)**	**N**	**Mean (SD)**
Age at ultrasound assessment (years)	3035	17.8 (0.4)	1101	17.9 (0.4)
AUDIT score	2555	6.9 (4.6)	897	6.9 (4.8)
MVPA (mins/day)	1216	24.2 (18.8)	396	22.8 (17.0)
BMI (kg/m ^2^)	3035	22.7 (3.9)	1101	23.0 (4.0)
Energy intake (kcal)	2521	1968.0 (506.2)	844	1953.2 (504.6)

N represents the maximum sample size based on those with complete data for ALT/AST or USS, age, sex, BMI and at least one metabolic trait. Abbreviations: ALT: alanine aminotransferase; AST: aspartate aminotransferase; USS: ultrasound scan; AUDIT: alcohol use disorders identification test; MVPA: moderate to vigorous physical activity; BMI: body mass index; SD: standard deviation.

### Prevalence of NAFLD by different methods of assessment

**Figure 3.**
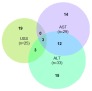
Comparison of three different indicators of NAFLD. These analyses were undertaken in 1,088 participants with data that allowed all three indicators of NAFLD to be applied. USS (ultrasound scan) defined steatosis is evidence of steatosis on ultrasound scan; elevated AST (aspartate aminotransferase) is anyone with AST >40 U/l; elevated ALT (alanine aminotransferase) is anyone with ALT >40 U/l.

The prevalence of NAFLD was similar across the three different indicators (2.3%, 3.1% and 2.3%, for USS steatosis, elevated ALT (>40 U/l) and elevated AST (>40 U/l), respectively), but with varying levels of overlap in cases across the measures. Of the 1,088 individuals who had data for all three indicators of NAFLD, only three met all three criteria (0.3%), with overlap between elevated ALT and elevated AST being greatest. In this sample there was no overlap between USS steatosis and elevated AST ( Figure 3). As we might have expected, the prevalence of obesity (BMI > 30kg/m ^2^) was greater in those with each indicator of NAFLD, but was greatest for USS steatosis (60%, 20% and 12% for USS steatosis, elevated ALT and elevated AST, respectively, compared to 5% for participants without these indicators).

### Metabolic profile of NAFLD

**Figure 4.**
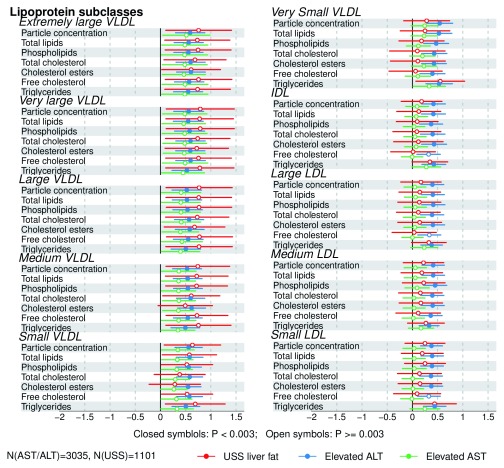
Multivariable associations between elevated ALT, elevated AST and ultrasound-assessed steatosis (USS steatosis) and standardized metabolic traits. Associations were adjusted for age, sex and BMI. Points represent the difference in mean metabolic trait concentrations in standard deviation units for those with NAFLD compared to those without NAFLD (for each of the three indicators). Horizontal coloured lines represent 95% confidence intervals. Abbreviations: VLDL: very low density lipoprotein; IDL: intermediate density lipoprotein; LDL: low density lipoprotein; BMI: body mass index.

Figure 4– Figure 6 present the associations between NAFLD, assessed by USS, elevated ALT and elevated AST and each of the 154 metabolic traits in SD units, adjusted for age, sex and BMI. These results are tabulated in full in Extended data: Table 1–Extended data: Table 3, and in original units (mostly μmol/l) in Extended data: Table 4–Extended data: Table 6. Extended data: Figure 1–Extended data: Figure 3 show the same results comparing both model 1 (age- and sex-adjusted) and model 2 (age-, sex- and BMI-adjusted); these show that adjustment for BMI did attenuate many associations. The associations between each indicator of NAFLD and the VLDL metabolic traits were most attenuated after adjustment for BMI, with the effect of greatest magnitude for USS steatosis (attenuated by approximately 50%) and the weakest magnitude for elevated AST. However, even with this adjustment there were wide-spread associations of NAFLD with multiple metabolic traits, and here we focus on the results with adjustment for BMI.

**Figure 5.**
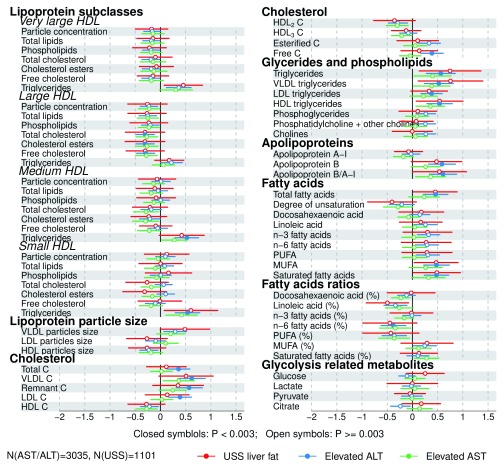
Multivariable associations between elevated ALT, elevated AST and ultrasound-assessed steatosis (USS steatosis) and standardized metabolic traits. Associations were adjusted for age, sex and BMI. Points represent the difference in mean metabolic trait concentrations in standard deviation units for those with NAFLD compared to those without NAFLD (for each of the three indicators). Horizontal coloured lines represent 95% confidence intervals. Abbreviations: HDL: high density lipoprotein; C: cholesterol; MUFA: monounsaturated fatty acid; PUFA: polyunsaturated fatty acid; BMI: body mass index.

**Figure 6.**
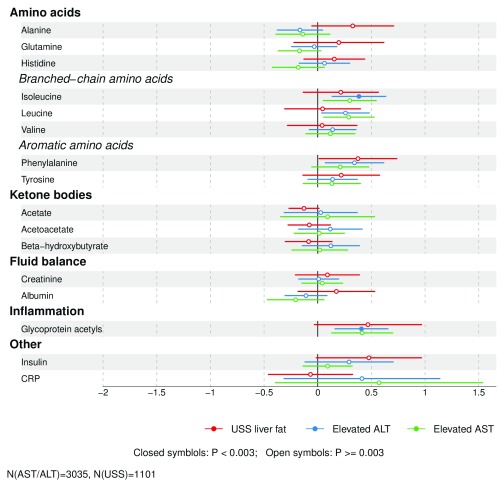
Multivariable associations between elevated ALT, elevated AST and ultrasound-assessed steatosis (USS steatosis) and standardized metabolic traits. Associations were adjusted for age, sex and BMI. Points represent the difference in mean metabolic trait concentrations in standard deviation units for those with NAFLD compared to those without NAFLD (for each of the three indicators). Horizontal coloured lines represent 95% confidence intervals. Abbreviations: CRP: C-reactive protein; BMI: body mass index.

***Lipoprotein subclasses.*** All three indicators of NAFLD were associated with higher concentrations of the extremely large, very large, large, medium and small VLDL metabolic traits. The largest effect sizes were observed between USS steatosis and the extremely large, very large and large VLDLs, with effect sizes ranging from 0.6 to 0.8SD. Elevated ALT was positively related to very small VLDL, IDL, and large, medium and small LDL metabolic traits. Effect estimates for USS steatosis and elevated AST were closer to the null, apart from triglycerides in these particles, for which positive associations of a similar magnitude were observed for all three indicators. There was evidence of consistent (across all three indicators) negative associations between NAFLD and very large and large HDL metabolic traits, except for triglycerides in these particles. Associations between all indicators of NAFLD and medium and small HDL metabolic traits were weakly negative or null, again except triglycerides in medium and small HDL, for which positive associations were detected. All three indicators were positively associated with VLDL particle size and negatively associated with HDL particle size. We found no strong evidence for an association between any indicator of NAFLD and LDL particle size.

***Cholesterol and triglycerides.*** Elevated ALT was positively associated with most cholesterol measures, except for the HDLc metabolic traits (HDLc, HDL _2_c and HDL _3_c), for which negative or null associations were observed. USS steatosis and elevated AST were also positively associated with VLDL and remnant cholesterol and negatively associated with HDLc and HDL _2_c. Elevated AST was additionally negatively associated with HDL _3_c. Standardized mean concentrations of all triglycerides were higher in those with NAFLD.

***Fatty acids.*** Total fatty acid concentrations were higher in individuals with NAFLD, with somewhat larger differences for USS steatosis and elevated ALT: effect sizes for USS steatosis and elevated ALT were 0.45SD and 0.46SD, respectively, compared to 0.23SD for elevated AST. There was some evidence for a decreased degree of unsaturation in individuals with NAFLD. Monounsaturated and saturated fatty acid concentrations were also higher in individuals with NAFLD, although evidence for a positive association with percentage of total fatty acids was weaker. Linoleic acid, omega-3 (n3), omega-6 (n6) and polyunsaturated (PUFA) fatty acid concentrations were higher in those with USS steatosis and elevated ALT, but ratio of these fatty acids to total fatty acids showed negative (linoleic acid, omega-6, PUFA) or null (omega-3) associations with USS steatosis and elevated ALT.

***Low molecular weight metabolites.*** Of the low molecular weight metabolites, concentrations of the branched-chain and aromatic amino acids and the inflammatory marker glycoprotein acetyls were higher in those with NAFLD, except for leucine and valine in individuals with USS steatosis, for which there was no difference in concentration.

**Figure 7.**
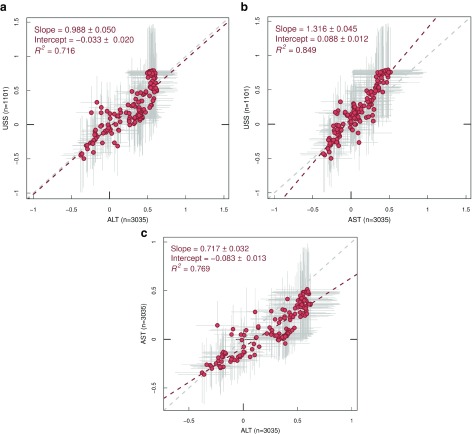
Correspondence between indicators of NAFLD. Each point represents a metabolic trait and the position represents the standardized mean difference for those with NAFLD as indicated by USS steatosis (( **a**) and ( **b**)) or elevated AST ( **c**) on the y-axis and those with NAFLD indicated by elevated ALT (( **a**) and ( **c**)) or elevated AST ( **b**) on the x-axis. These estimates were adjusted for age, sex and BMI. Horizontal grey lines represent confidence intervals for x-axis associations, and vertical grey lines represent confidence intervals for y-axis associations. The red dashed line represents the regression line between x-axis and y-axis associations for each metabolic trait. R ^2^ indicates goodness-of-fit. A slope of 1 with an intercept of 0 (grey dashed line) and all dots sitting on that line (R ^2^ = 1) would indicate exact correspondence, in terms of direction and magnitude, between associations of the pair of indicators of NAFLD with metabolic traits. Abbreviations: USS: ultrasound scan; ALT: alanine aminotransferase; AST: aspartate aminotransferase.

***Magnitude of associations.*** Although some magnitudes of associations varied slightly between the three indicators of NAFLD, and *p*-values differed due to different sample sizes (smaller for USS), overall there was a high level of consistency between the three indicators of NAFLD in their associations with the metabolic traits, with the goodness of fit between each pair (measured using R ^2^) all greater than 0.7 ( Figure 7). Furthermore, where associations were observed these were large, usually in the range of 0.4 to 0.7 SD difference between those with and without NAFLD.

### Additional analyses

There was almost perfect correspondence between the age, sex and BMI, and the age, sex and TBFM–adjusted models (R ^2^>0.99; Extended data: Figure 4). Results were the same when individuals with diabetes were excluded and were virtually the same when extreme metabolic trait outliers were removed, the one exception being an inverse association of elevated ALT and elevated AST with acetate when outliers were removed (data available on request). Results were also consistent when all indicators were compared to a consistent group without any of the three indicators of NAFLD (Extended data: Figure 5).

**Figure 8.**
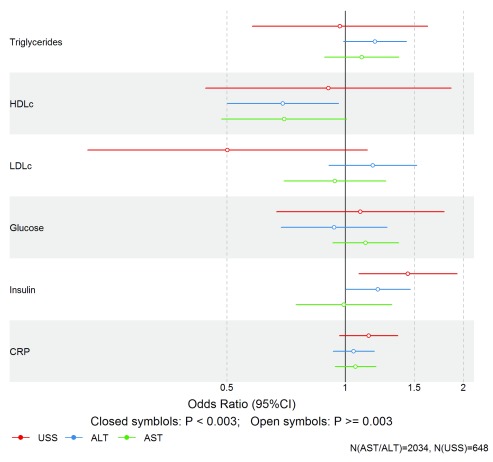
prospective associations between enzyme-assessed cardiometabolic risk factors at average age 15 and indicators of NAFLD at average age 17. Associations were adjusted for age, sex and BMI. Points represent the odds ratio for NAFLD (for each of the three indicators) per standard deviation increase in cardiometabolic risk factor concentration. Bars represent 95% confidence intervals. Abbreviations: HDLc: high density lipoprotein cholesterol; LDLc: low density lipoprotein cholesterol; ALT: alanine aminotransferase; AST: aspartate aminotransferase; USS: ultrasound scan.

Results were largely the same between NMR- and enzyme-assessed total triglycerides, HDLc, LDLc and glucose (Extended data: Figure 6). USS steatosis and elevated ALT were positively associated with insulin, and elevated ALT and AST positively associated with CRP, although these were imprecisely estimated with wide CIs ( Figure 6). We found no strong evidence that enzyme-assessed triglycerides, LDLc or glucose concentrations at age 15 were associated with subsequent NAFLD at age 17; HDLc at 15 was inversely associated with NAFLD at 17 and insulin at 15 was positively associated with odds of USS steatosis and elevated ALT at 17 ( Figure 8).

The NAFLD related genetic variant, rs738409 C>G SNP (encoding PNPLA3 I148M), was positively associated with USS NAFLD (OR=1.74 [0.86, 3.48]) and more weakly with elevated ALT (OR=1.30 [0.88, 1.93]) but not notably with AST (OR=1.03 [0.63, 1.66]). As expected, these associations are imprecise with wide confidence intervals. The magnitude of the point estimates are consistent with our USS measure being a valid measure of NAFLD and AST being a less specific indicator; they do not support AST as a specific marker of NAFLD.

## Discussion

We have found evidence for a disrupted metabolic profile in adolescents with evidence of NAFLD, including consistent associations of NAFLD, using three different indicators, with an adverse lipoprotein and lipid profile. This adverse lipid and lipoprotein profile was characterized by increased concentrations of all metabolic traits in the extremely large to small VLDL subclasses, all triglycerides, remnant cholesterol and saturated fatty acids. We also found increased concentrations of glycoprotein acetyls, which are considered to be a stable marker of chronic inflammation ^43^ and have been shown to associate with cardiovascular disease in several independent adult cohorts ^29^. These associations were present with adjustment for general adiposity by either BMI or DXA-determined TBFM. Magnitudes of association were generally larger for USS steatosis, particularly for the larger VLDL metabolic traits, though given the smaller sample size for analyses with USS steatosis, the confidence intervals were wider. A possible stronger association with USS steatosis may reflect the fact that it is likely to be measuring more severe NAFLD ^44^.

To the best of our knowledge, this is the first study to determine the metabolic profile of NAFLD in a large population of apparently healthy adolescents. Our results highlight the possibility that NAFLD at this young age is potentially already having adverse consequences that might increase the future risk of diabetes and cardiovascular disease. Our findings have some consistency with findings from an adult cohort (the Young Finns study) that used the same NMR platform, including strong positive associations with extremely large to small VLDL particle concentrations and glycoprotein acetyls ^20^. In contrast to our study, USS steatosis in that adult cohort was also associated with small HDL particle concentration, CRP, glucose, lactate and pyruvate, and the magnitudes of associations with amino acids were stronger than we found. Associations with a wider-range of metabolic traits in the adult cohort might reflect a greater accumulation of NAFLD risk factors and longer exposure to NAFLD with greater age, but this requires further analyses in independent cohorts with repeat metabolomic measurements at different ages.

The Young Finns study, although mostly based on cross-sectional analyses, examined associations of metabolic profiles with subsequent occurrence of USS steatosis. In contrast, given the role of the liver in multiple metabolic pathways, we hypothesized that accumulation of liver fat would precede any alterations in metabolic profile and we therefore treated NAFLD as the exposure in our analyses. As our analyses were cross-sectional, we are unable to assess temporality. However, we found little evidence that enzyme-assessed fasting total triglycerides, LDLc, glucose or CRP at age 15 were associated with subsequent NAFLD at 17, providing evidence for liver fat accumulation preceding metabolic disruption. The largest effect sizes we observed were for the VLDL traits; given the role of the liver in assembly and secretion of VLDLs, our hypothesized causal direction is further justified.

The strengths of this study are its large sample size, the ability to adjust for BMI or fat mass and the exploration of a wide range of metabolic traits using an NMR platform that is highly reproducible ^30^. An additional strength of this study is the inclusion of three different measures of NAFLD, USS and two different liver enzymes ALT and AST, providing stronger evidence for associations between NAFLD and related metabolic traits. Although USS steatosis is the most commonly used measure of NAFLD in epidemiological studies ^45^, our sample size was increased when using ALT or AST as a measure of NAFLD, providing more power to detect associations. Our genetic analyses provide some support for USS and elevated ALT as markers of NAFLD.

Liver biopsy is considered the gold-standard methodology to diagnose NAFLD but would be unethical to use in a population of healthy adolescents. Liver biopsy may also miss liver fat because of sampling variability, as only a small sample of liver is selected ^46^. The EASL-EASD-EASO guidelines recommend USS for the diagnosis of steatosis but a biopsy is required for the presence of NASH ^47^. Therefore, our study is unable to draw any conclusions about differences in metabolic profiles of those with NASH. Reliability and accuracy of USS, compared to biopsy, has been demonstrated for moderate to severe fatty liver ^48^. However, USS assessment of steatosis is less sensitive when liver fat accumulation is <30% ^44^ and in obese individuals ^49^, who have a greater risk of developing NAFLD ^2, 10^, yet our results were consistent across three different indicators of NAFLD and we adjusted for BMI or TBFM in our regression model. There is currently no consensus for the ALT/AST threshold to indicate NAFLD. A systematic review of prevalence of NAFLD in children and adolescents identified that ALT was by far the most common enzyme used to identify NAFLD, with a threshold of 40U/l most commonly used. AST was used in just one of the studies, again with a threshold of 40U/l used to identify NAFLD ^2^. We are not aware of a study comparing the sensitivity or specificity of this threshold to biopsy-diagnosed disease in a general population as here. Our population was 98% white British, limiting generalizability to adolescents of other ethnicities. Some of our results had wide CIs (particularly the associations between USS steatosis and the metabolic traits) and although our findings were mostly consistent with those from one previous study in adults, we are not aware of any other study with data on NAFLD and a wide range of metabolic traits that could be used to replicate our findings in healthy adolescents.

In conclusion, this study provides evidence that NAFLD in adolescence is already associated with widespread adverse lipid and fatty acid profiles, and with the inflammatory biomarker glycoprotein acetyls. These findings are important as they imply adverse profiles, that in adults are related to risk of cardiovascular disease ^29^, are apparent in early life. Follow-up data collection in this population will allow prospective analysis to determine if these differences persist into adulthood, which would further imply their potential role in mediating the impact of adolescent NAFLD on future adverse cardiovascular disease. Thus, our findings provide the foundations for further research exploring causal relations between adolescent NAFLD and widespread metabolic disruption, that potentially persists and could result in increased cardiovascular risk.

## Data availability

### Underlying data

ALSPAC data access is through a system of managed open access. The steps below highlight how to apply for access to the data included in this data note and all other ALSPAC data. The datasets presented in this article are linked to ALSPAC project number B1032, please quote this project number during your application. The ALSPAC variable codes highlighted in the dataset descriptions can be used to specify required variables.

1. Please read the ALSPAC access policy (PDF, 627kB) which describes the process of accessing the data and samples in detail, and outlines the costs associated with doing so.

2. You may also find it useful to browse our fully searchable research proposals database, which lists all research projects that have been approved since April 2011.

3. Please submit your research proposal for consideration by the ALSPAC Executive Committee. You will receive a response within 10 working days to advise you whether your proposal has been approved.

If you have any questions about accessing data, please email alspac-data@bristol.ac.uk.

The ALSPAC data management plan describes in detail the policy regarding data sharing, which is through a system of managed open access.

The study website also contains details of all the data that is available through a fully searchable data dictionary: http://www.bristol.ac.uk/alspac/researchers/data-access/data-dictionary/.

### Extended data

Extended data table and figures are available via the Open Science Framework: Metabolic profiling of adolescent non-alcoholic fatty liver disease, https://doi.org/10.17605/OSF.IO/SNA6C ^50^.

Data are available under the terms of the Creative Commons Zero “No rights reserved” data waiver (CC0 1.0 Public domain dedication).

Table 1. Age, sex and BMI adjusted associations between USS-assessed steatosis and metabolic traits in standard deviation units.

Table 2. Age, sex and BMI adjusted associations between elevated ALT and metabolic traits in standard deviation units.

Table 3. Age, sex and BMI adjusted associations between elevated AST and metabolic traits in standard deviation units.

Table 4. Age, sex and BMI adjusted associations between USS-assessed steatosis and metabolic traits in molar concentrations.

Table 5. Age, sex and BMI adjusted associations between elevated ALT and metabolic traits in molar concentrations.

Table 6. Age, sex and BMI adjusted associations between elevated AST and metabolic traits in molar concentrations.

Figure 1. Multivariable associations between USS-assessed steatosis and 154 metabolic traits.

Figure 2. Multivariable associations between elevated ALT and 154 metabolic traits.

Figure 3. Multivariable associations between elevated AST and 154 metabolic traits.

Figure 4. Correspondence between models adjusted for age, sex and BMI and models adjusted for age, sex and total body fat mass (TBFM) for each indicator of NAFLD.

Figure 5. Correspondence between results of analyses where the control group was indicator specific (i.e. did not meet the criteria for NAFLD based on one indicator only) and results where the control group did not meet the criteria for NAFLD based on all three indicators.

Figure 6. Comparison of multivariable associations between indicators of NAFLD, and non-NMR-assessed and NMR-assessed cardiometabolic risk factors.
