# Supplementary material for: Metabolic profiling of adolescent non-alcoholic fatty liver disease
Source: Wellcome Open Res. 2019 Sep 19;3:166. Originally published 2018 Dec 28. [Version 2] doi: 10.12688/wellcomeopenres.14974.2 (PMC6338132; doi:10.12688/wellcomeopenres.14974.2)
Supplement: Peer review history

# Peer review: Reviewer response for version 2

**DOI:** 10.21956/wellcomeopenres.16921.r36509

**Published:** 2019-09-19

**Authors:** Stefan Stender

**Affiliations:** 1 Department of Clinical Biochemistry, Rigshospitalet, Copenhagen University Hospital, Copenhagen, Denmark

I confirm that I have read this submission and believe that I have an appropriate level of expertise to confirm that it is of an acceptable scientific standard.

# Peer review: Reviewer response for version 1

**DOI:** 10.21956/wellcomeopenres.16333.r34503

**Published:** 2019-01-14

**Authors:** Stefan Stender

**Affiliations:** 1 Department of Clinical Biochemistry, Rigshospitalet, Copenhagen University Hospital, Copenhagen, Denmark

**Summary**

 Hartley and colleagues report associations between NAFLD and 154 different plasma metabolites (mainly lipoproteins and amino acids) among 3048 children and adolelescents from the ALSPAC cohort. NAFLD was assessed by 1) ultrasound, 2) elevated alanine aminotransferase levels, or 3) elevated aspartate aminotransferase levels. The main finding of the study was that NAFLD was associated with pervasive changes in plasma metabolites, with the most robust effect seen for plasma VLDL.

**Strengths**

 1) The metabolic characterization is comprehensive.

 2) The statistical methods are meticulous and state-of-the art.

 3) There are few large cohorts of well-characterized children and adolescents with measurements of NAFLD available. The study adds substantially to the NAFLD literature. 

 4) It is interesting (and new) to see the 'metabolic fingerprints' of ultrasound- vs ALT- vs AST-defined NAFLD.

**Weaknesses**

 1) The main weakness relates to the use of nonspecific and nonsensitive methods to assess NAFLD. Ultrasound is a relatively imprecise method to assess liver fat with, especially in those with low levels of liver fat (like almost all individuals in the present study). Elevated ALT and/or AST also have low sensitivity and specificity for detecting NAFLD. This limitation is acknowledged in the paper.

 One way of addressing this weakness could be to show the association of the 3 NAFLD-phenotypes with PNPLA3 I148M, a strong and specific genetic risk factor for NAFLD. If US-, ALT, and AST-defined NAFLD truly reflects NAFLD in this cohort, they should also associate with PNPLA3 I148M. In other words PNPLA3 I148M could be used a 'genetic positive control'.

I confirm that I have read this submission and believe that I have an appropriate level of expertise to confirm that it is of an acceptable scientific standard.

# Response

**Published:** 2019-09-09

**Authors:** April Hartley

**Affiliations:** UNIVERSITY OF BRISTOL, UK

We thank Dr Stender for his suggestions. We have determined the association between the rs738409 C>G SNP (encoding PNPLA3 I148M) in our study population and present and discuss these results in the revised paper. Bearing in mind the same sample sizes for these analyses (794 for USS and 2,242 for ALT/AST), and that this variant only explains 2.4% of the variance in CT-assessed steatosis (Speliotes *et al*, PLOS Genetics, 2011), we believe our findings support USS as a more specific marker of NAFLD than ALT with little evidence that AST is a marker.

# Peer review: Reviewer response for version 1

**DOI:** 10.21956/wellcomeopenres.16333.r34501

**Published:** 2019-01-07

**Authors:** Warwick B. Dunn

**Affiliations:** 1 School of Biosciences, University of Birmingham, Birmingham, UK

The manuscript submitted details a NMR-based metabolic profiling study of NAFLD in adolescents of approximate age 17. The manuscript was descriptively written to allow the reader to understand the methods applied and to be able to repeat the work and was also written to easily understand the major conclusions.

 The strengths of the manuscript are as follows:

 1. The cohort studied (ALSPAC) was highly appropriate as the recruited subjects are highly characterised and phenotyped at different ages. This cohort also provides the possibility for subject follow-up in the future.

 2. The study of NAFLD in young adults is novel and innovative. NAFLD can develop and remain as a human ages or can develop in to more serious diseases. The ability to stratify subjects at early stages of NAFLD and provide follow-up in 5 and 10 years time will be innovative and provide more information on how NAFLD develops in to more serious conditions.

 3. The metabolic profiling approach used were highly appropriate and is highly proven in these types of studies. The approach provides data on different aspects of metabolism including lipoproteins, lipid metabolism and branched chain amino acids; all of these have been defined as important in human ageing and disease. All data is also quantitative and so is comparable to other datasets which were acquired applying the same approach.

 4. The results clearly show the association between early NAFLD and many of the 154 metabolic traits measured and raises some interesting questions to be studied further in follow-up.

 The weaknesses of the manuscript are as follows:

 1. The use of two liver enzymes as markers of NAFLD was interesting as these enzymes are not specific to NAFLD but are more general markers of liver health/disease and are associated with different liver diseases. Inclusion of a discussion as to why these two measures were included and why they differ in magnitude to steatosis measures would have benefited the manuscript. Construction of the manuscript with a focus on ultrasound steatosis measures would have allowed easier understanding.

 2. Replication in an independent cohort would have increased the confidence in the generalisability of the conclusions provided, though I am unaware of a cohort to be able to apply for replication.

 3. The metabolic profiling approach of NMR spectroscopy is a strength for understanding specific areas of metabolism but the lack of inclusion of other metabolic profiling approaches to complement the NMR data is a weakness and should be considered for future work. The inclusion of UPLC-MS to investigate the same plasma samples would be appropriate to provide information on a wide number of metabolites and metabolic pathways; for example, lipidomic analysis would provide greater information on the individual lipids in each lipid class whereas NMR only provides a single measure which integrates all of the separate lipids.

 Overall, the manuscript provides robust data and biological conclusions and opens up some interesting biological questions requiring further study in the future.

I confirm that I have read this submission and believe that I have an appropriate level of expertise to confirm that it is of an acceptable scientific standard.

# Response

**Published:** 2019-09-09

**Authors:** April Hartley

**Affiliations:** UNIVERSITY OF BRISTOL, UK

We thank Professor Dunn for the detailed review of our manuscript and apologise for the delay in responding to the suggestions to improve the manuscript.

We thank Prof. Dunn for the suggestion of constructing the manuscript with a focus on USS steatosis. We agree that clarifying the use of the liver enzymes would be valuable. However, we do not wish to change the focus of the paper to solely the USS outcomes. First, we prefer to maintain all results from our analysis plan in the paper. Second ALT and AST are routinely collected in large population-based studies and elevated ALT is commonly used in epidemiological studies, e.g. to assess prevalence (Anderson *et al*, PLoS One, 2015) or potential risk factors (De Silva *et al*, Diabetes, 2019). Third, as we only observed 25 cases of USS-steatosis in our population and the outcome measure was binary, our confidence intervals were wide for most of the associations with USS, overlapping the null. Presenting the ALT, AST and USS results allows us to contrast the magnitude of effect for the more specific measure (USS) and the less specific biomarkers of NAFLD (ALT, AST). We have revised the discussion to address this.

We also agree that exploring UPLC-MS lipidomic data would be an interesting and informative follow-up to the current study. Unfortunately, these data are not currently available in our studies.
